# The Use of 3D Titanium Miniplates in Surgical Treatment of Patients with Condylar Fractures

**DOI:** 10.3390/jcm9092923

**Published:** 2020-09-10

**Authors:** Maciej Sikora, Maciej Chęciński, Marcin Sielski, Dariusz Chlubek

**Affiliations:** 1 Department of Maxillofacial Surgery, Hospital of the Ministry of Interior, Wojska Polskiego 51, 25-375 Kielce, Poland; sikora-maciej@wp.pl (M.S.); marcinsielski@gazeta.pl (M.S.); 2 Department of Biochemistry and Medical Chemistry, Pomeranian Medical University, Powstańców Wlkp. 72, 70-111 Szczecin, Poland; 3 STOMADENT Non-public Healthcare Institution, Dental Clinic, Kościuszki 32, 46-320 Praszka, Poland; maciej@checinscy.pl

**Keywords:** 3D miniplates, condylar fractures, internal fixation, mandibular condyle fractures, open reduction

## Abstract

The aim of this study was to evaluate the effectiveness of open treatment of mandibular condyle fractures using 3D miniplates. A group of 113 patients has been chosen for evaluation, including 100 men and 13 women. After hospitalization, each patient underwent a 6-month postoperative follow-up. The material chosen for the analysis consisted of data collected during the patient’s stay in the hospital as well as the postoperative outpatient care. A single 4-hole Delta Condyle Compression Plate (4-DCCP) was used in 90 out of 113 (79.6%) cases. In 16 out of 113 (14.2%) patients, the Trapezoid Condyle Plate (4-TCP or 9-TCP) was used. The remaining cases required more than one miniplate. No 3D miniplate fractures were found in the study subjects during the analyzed observation period. Loosening of one or more osteosynthesis screws was observed in 4 out of 113 (3.5%) patients. Screw loosening was a complication that did not affect bone healing in any of the patient cases. The conducted research confirms that titanium 3D mini-plates are easy to adjust and take up little space, therefore they can easily be used in cases of mandibular condyle base and lower condyle neck fractures. The stability of the three-dimensional miniplates for osteosynthesis gives very good reliability for the rigid fixation of the fractured mandibular condyle.

## 1. Introduction

Mandibular condyle fractures are a heterogeneous group of fractures which classification and methods of therapy are constantly improving [1,2,3]. According to various studies, the incidence of condylar fractures among all mandibular fractures is 23% to 35% [4,5]. Means of treating condylar fractures can be broken down into closed and open treatment methods. All treatment strategies listed include subsequent functional therapy, and each of them can be supplemented with maxillo-mandibular fixation [6,7,8]. Despite creating a risk of injury to the facial nerve with surgical access the closed treatment approach is not exempt from disadvantages having less functional results in comparison [9]. There are also voices indicating that both strategies (closed treatment vs. open treatment) are not easily comparable [10].

Recent years have brought clear guidelines indicating appropriate surgical approaches depending on the location of the fracture gap [1,2,3,11]. Materials that are typically used for assessing base of the condyle and lower part of the mandible neck fractures are miniplates fixed to the bone surface with screws [2,12,13]. The most common types are straight plates with linear arrangement of holes, L-shaped plates and multidimensional plates (3D plates) [7,14]. It has been proved that use of a single straight or L-shaped plate presents less satisfactory mechanical properties than using of two plates (double plate technique) or a single 3D plate [14,15]. These include Strut, Rhombic, Trapezoid (TCP), Delta (DCCP), Lambda, A-shape (ACP) and X-shape (XCP) patterns [7,16,17,18]. There are also miniplates made of resorbable materials (e.g., PLLA, PDLLA), but their use is still not common due to the lower mechanical strength than that presented by their metal counterparts [19,20]. In the search for the most suitable materials for making 3D miniplates, numerous in vitro and animal tests are carried out, focusing mainly on areas of mechanical strength and body response [17,21,22,23]. The future solution may be fixation elements prepared for an individual patient [24,25].

## 2. Aim of the Study

This study was designed to assess the effectiveness of open treatment of mandibular condyle fractures using 3D miniplates. The paper summarizes a prospective cohort observational study at one institution.

## 3. Materials and Methods

This study was conducted in the Department of Maxillofacial Surgery, Hospital of the Ministry of Interior in Kielce (Poland), in accordance with the Declaration of Helsinki. All procedures involving human subjects were approved by the Ethics Committee of the Pomeranian Medical University in Szczecin (Approval No. KB-0012/43/06/2020/Z). Written informed consent was obtained from all subjects.

One hundred and thirteen patients (100 men and 13 women) operated on during the years 2013–2019 were included in the study and qualified in accordance with the date of the beginning of the therapeutic process. To our knowledge, the study group presented in this article is the second largest group indexed in PubMed for 3D miniplates. After the hospitalization, each patient was subjected to 6 months postoperative observation, which was equal to the evaluation period. Data regarding the period of stay in the hospital and collected during postoperative ambulatory care was used as material for analysis.

Classification of the condylar fractures performed for the purposes of this study was conducted according to the Comprehensive AOCMF Classification System [2] which derives from the earlier division of Loukota et al. [3].

The diagnosis in each case in the study group was based on a clinical examination and radiographs in panoramic and PA projections. Whenever these methods were insufficient, computed tomography (CT) scans were performed. The same diagnostic protocol was used postoperatively to control the position of the fragments. The value of three-dimensional diagnostics for full visualization of details is also appreciated by other authors [26].

Detailed criteria for inclusion to the study and exclusion from the study group are presented in Table 1 and Table 2.

Standard epidemiological data were collected for each patient during medical interview, clinical examination, hospitalization and postoperative ambulatory care. For this purpose, the form shown in Table 3 was used.

The open reduction and internal fixation (ORIF) surgery was performed under general anaesthesia. All fractures were rigidly fixed with standard three-dimensional (3D) Medartis Modus 2.0 plates. Detailed data on the 3D plates used is presented in Table 4.

During postoperative examination the maximum jaw opening was measured, the facial nerve function was assessed, the condition of the temporomandibular joints was examined, control X-rays were performed to assess bone healing and the state of the fixing material. The entire diagnostic and therapeutic process of patients was in accordance with current guidelines. Both surgery and all pre- and postoperative examinations were performed on patients in accordance with the standard procedure that is performed on patients with this type of injury. The form for collecting data during the operation and the postoperative observation period is presented in Table 5.

## 4. Results

**Figure 1:**
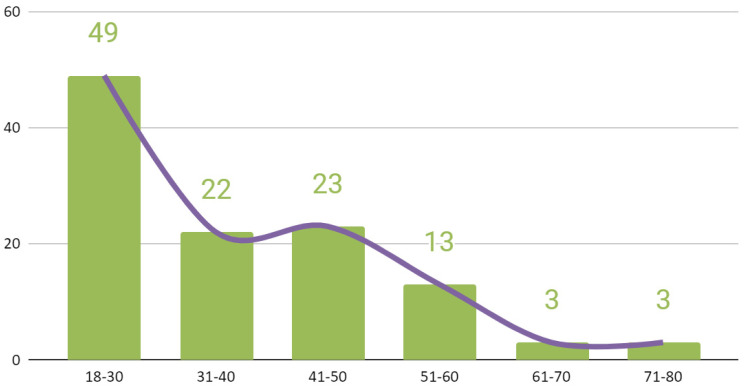
Classification of all patients in accordance to age range.

**Figure 2:**
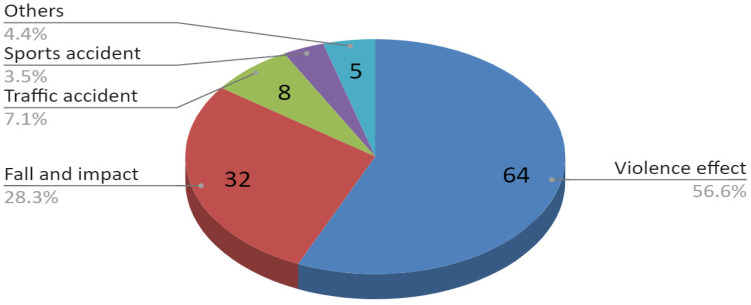
The causes of injuries for the entire study group.

**Figure 3:**
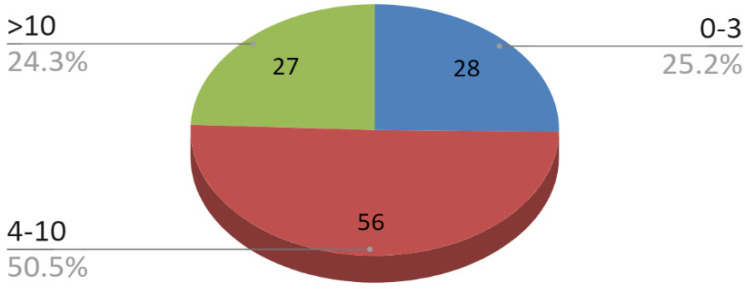
Time interval from trauma to surgery.

Patients were classified into six age categories (Figure 1) The causes of injuries for the entire study group are illustrated in Figure 2. Due to the more numerous male population, the distribution of injury causes for the whole group largely corresponds to the distribution of injury causes specified for the group of men. The time interval from trauma to surgery is shown in Figure 3. This time depended most on the time between trauma and admission to the maxillofacial surgery department. The delay in starting surgical treatment consisted mainly of delayed patient reporting to any medical facility and the procedure of transferring the patient to our hospital.

**Figure 4:**
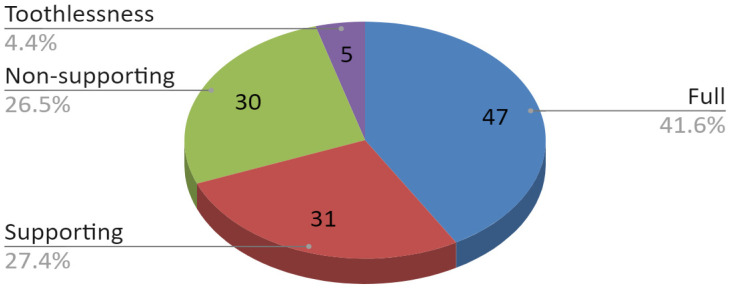
Dentition status of the patients.

**Figure 5:**
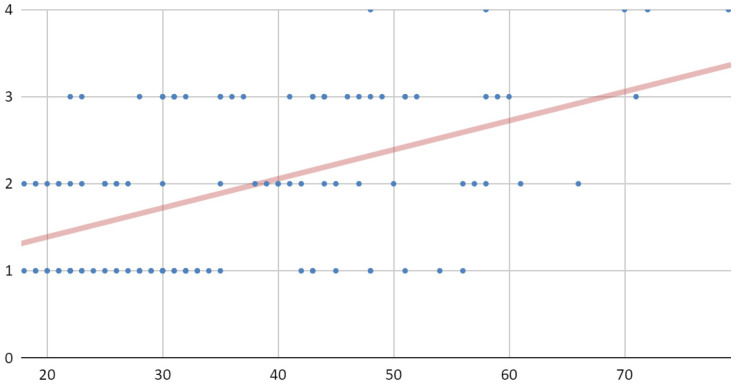
The correlation between the age of the patients and their dental condition. Description in the text.

The dentition status of the patients divided into four clinically significant categories is shown in Figure 4. Forty seven (47) of the 113 (41.6%) patients had full dental arches (1). Among patients with incomplete dental arches, three groups were distinguished: (2) with preserved support zones (supporting), (3) with partially or completely lost support zones (Non-supporting)—and (4) edentulous (toothlessness). The numbers in brackets correspond to these on the vertical axis of the graph depicted in Figure 5. This graph shows a correlation between the age of patients and their dental condition defined in the four categories mentioned above. The observed correlation takes the value of Pearson’s coefficient equal to 0.50.

**Figure 6:**
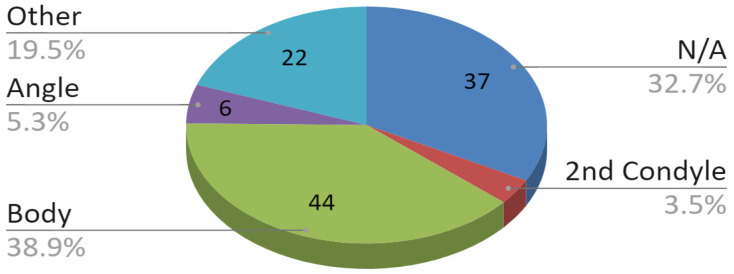
Concomitant fractures of the mandible.

The next data refers to the nature of the injury suffered. According to the pie chart in Figure 6, 76 of 113 (67.3%) patients tested had multiple mandible fractures. Thirty seven (37) out of the 113 (32.7%) subjects were diagnosed with isolated fractures of one condylar process. In the group of patients who suffered a multi-site fracture of the mandible, the majority of fractures covered two locations. In 50 of all 113 (44.2%) patients with condylar fracture, concomitant fractures included the body or the angle of the mandible. Bilateral fractures of the mandibular condyle processes without other coexisting fractures within this bone were rare. Such a fracture was reported only in 4 out of 113 (3.5%) patients. A group of 22 out of 113 (19.5%) subjects had a different combination of fractures, including concomitant alveolar fracture, mandibular branch fractures, and, most of all, complex fractures involving more than two locations.

**Figure 7:**
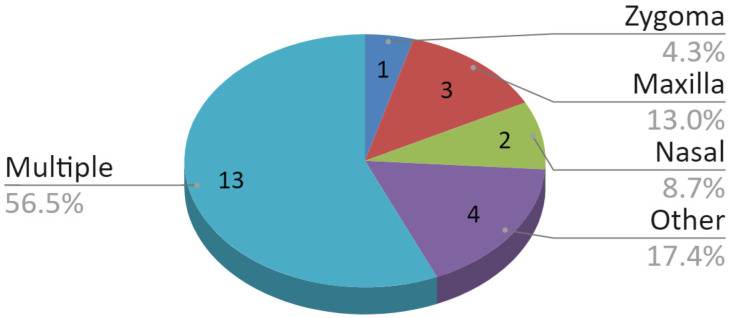
Division of coexisting non-mandibular bone fractures.

**Figure 8:**
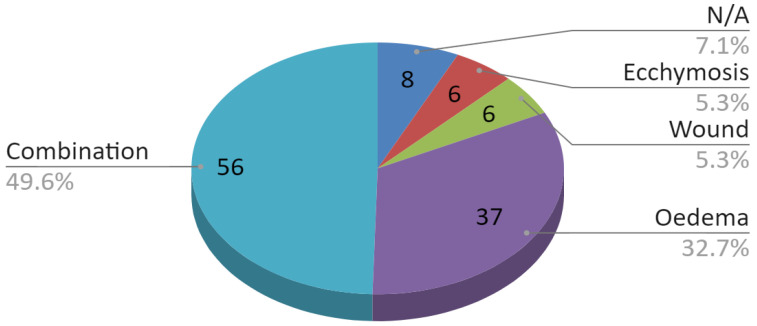
Coexisting soft tissue injuries.

About 80% of the fractures, which included the condylar process fracture, were solely in the mandible. The remaining 23 of 113 (20.4%) fractures involved more than one bone. A detailed division of coexisting non-mandibular bone fractures is shown in Figure 7. In this group, fractures of three or more bones predominated, i.e., mandible and at least two other bones. In turn, the pie chart in Figure 8 illustrates coexisting soft tissue injuries. In about half of the cases, they were composite injuries. Only in eight out of 113 (7.1%) examined patients no soft tissue injuries were found in physical examination.

Eighty eight (88) out of the 113 (77.9%) cases concerned the base of the condylar process according to the classification AOCMF Classification System [2]. The remaining 25 of 113 (22.1%) were low mandible neck fractures according to the same classification. As many as 99 out of 113 (87.6%) condylar fractures were displaced. About a quarter of all fractures involved mandibular head dislocation outside the acetabulum. This means that 30 of 99 (30.3%) displaced fractures were dislocated.

The information discussed below concerns the patient’s stay in the hospital ward. The vast majority (94.7%) of patients were operated using a retromandibular transparotid approach. In the remaining patients, surgeons decided to use intraoral (4.4%) and submandibular approaches (0.9%). In 90 of 113 (79.6%) cases, a single 4-hole Delta Condyle Compression Plate (4-DCCP) was used. In 16 of 113 (14.2%) patients it was decided to use a trapezoidal plate (4-TCP or 9-TCP). Seven out of 113 (6.2%) mandibular condylar fractures required the use of more than one mini-plate.

**Figure 9:**
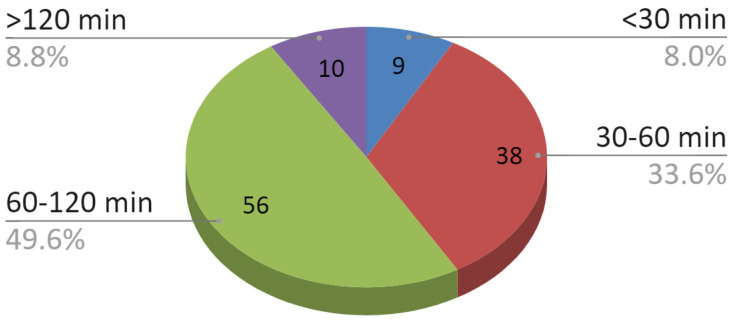
Duration of individual operations.

The time interval of the operation is shown in Figure 9. It was dependent, inter alia, on the extent of the injury. The vast majority of patients (68.1%) stayed in the ward from 4 to 10 days, 25.7% stayed for 1–3 days, longer hospitalizations (>10 days) concerned only seven out of 113 (6.2%) patients.

The following data reflects the results of the examination of patients 6 months after individual operations. According to the House and Brackmann scale [28], 106 out of 113 (93.5%) subjects recovered the full facial nerve function (I Grade). Out of the remaining seven patients, five (4.4%) showed a mild dysfunction of one or more of the facial nerve branches, noticeable on close inspection (Grade II). Obvious dysfunction of one or more branches of the facial nerve that did not lower the patient’s quality of life persisted 6 months after surgery in two out of 113 (1.8%) patients (Grade III).

**Figure 10:**
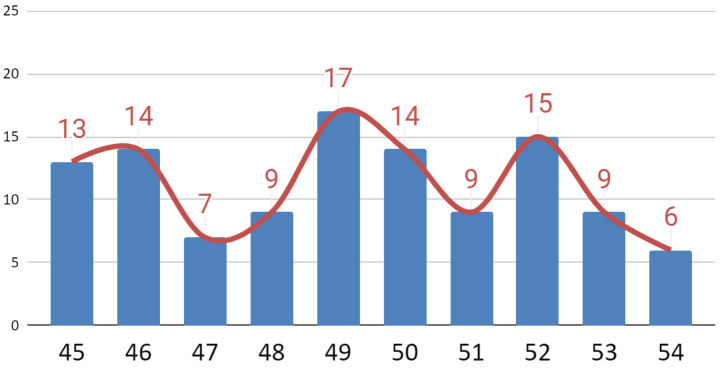
Distribution of patients depending on the amplitude of mouth opening.

The range of mandibular abduction was satisfactory for all patients. It was between 45 and 54 mm between the incisors of dental arches or dentures. This gave an average value of 49.23 mm with a standard deviation of 2.75 mm. The numbers of patients presenting individual mouth opening values are shown in Figure 10.

**Figure 11:**
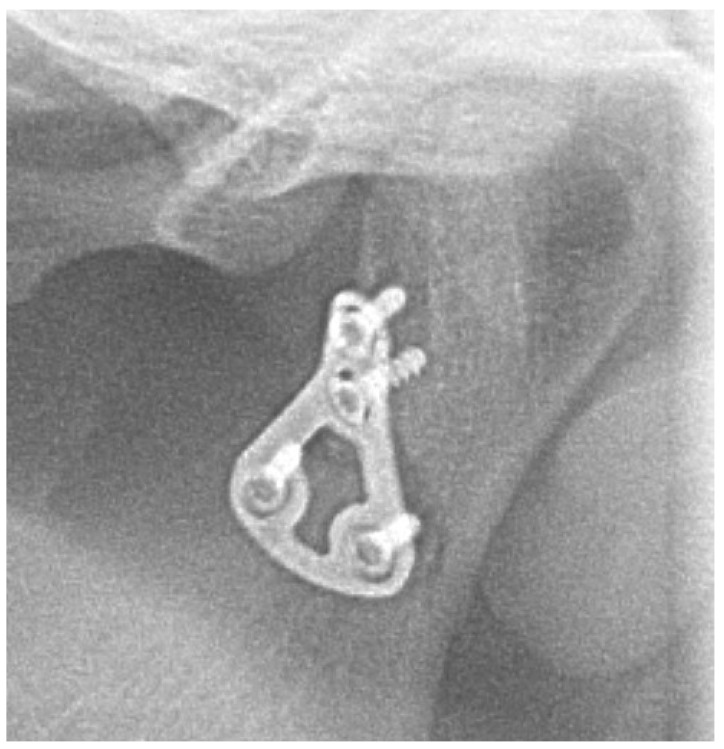
Patient A—An uncomplicated case—immediately after open reduction and internal fixation.

**Figure 12:**
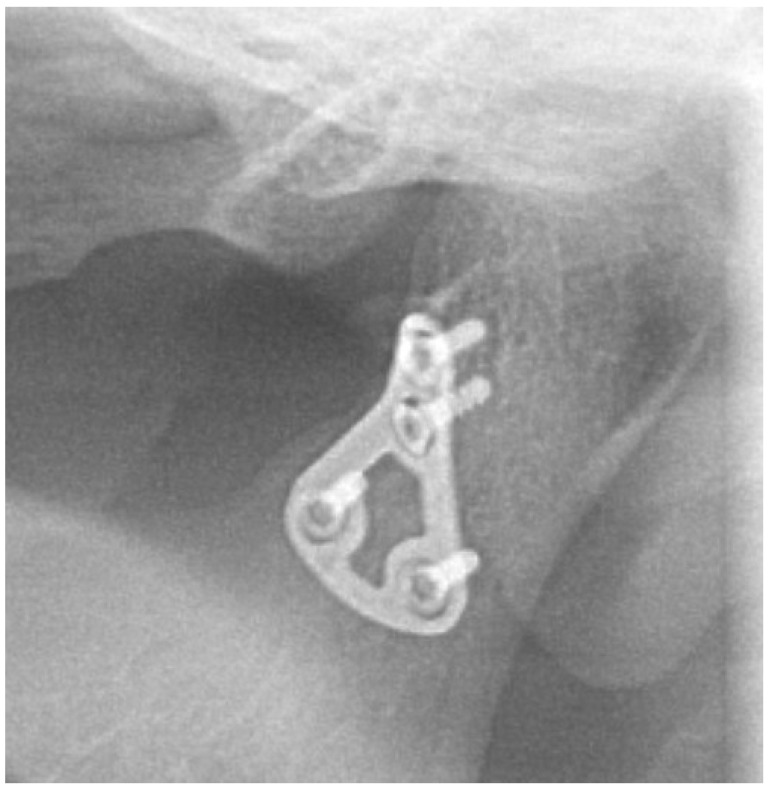
Patient A—An uncomplicated case—6 months after the surgery.

**Figure 13:**
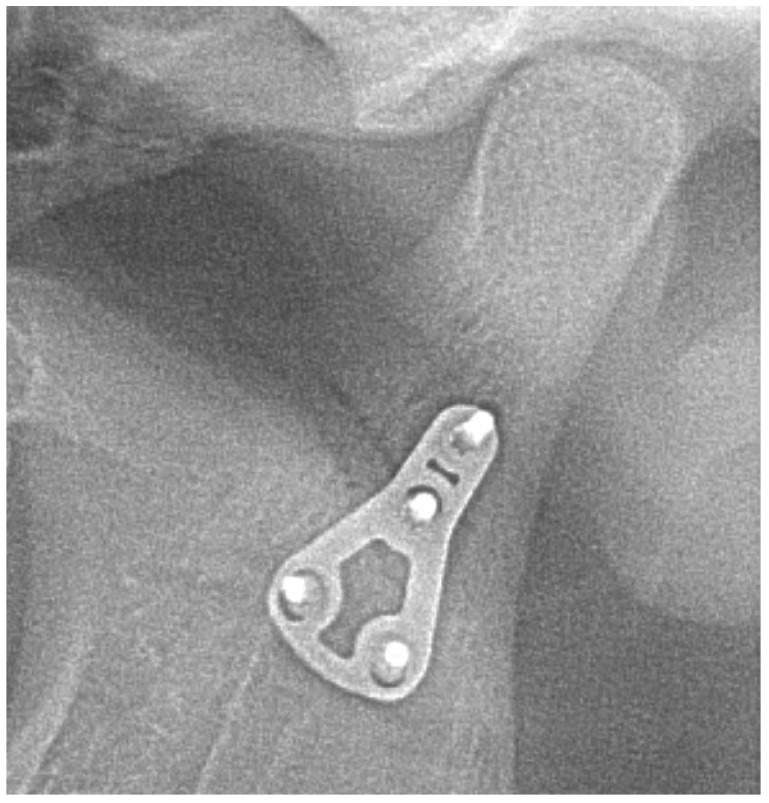
Patient B—A case complicated by screw loosening—immediately after open reduction and internal fixation.

**Figure 14:**
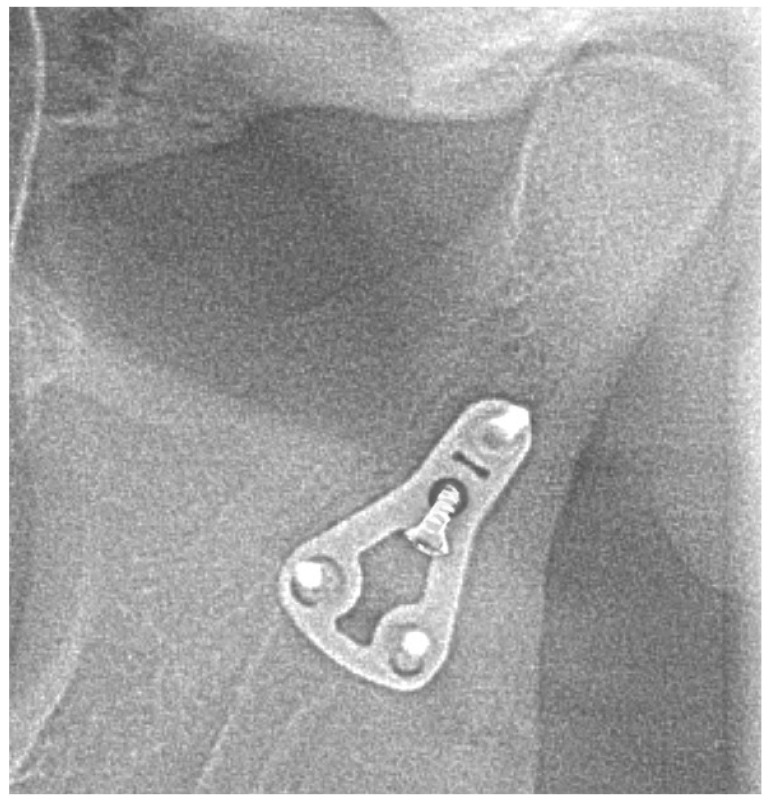
Patient B—A case complicated by screw loosening—6 months after the surgery.

No fractures of osteosynthesis plates were found in any of the cases. Therefore, the healing of bone fragments has not been disrupted in any of the cases. However, in four out of 113 (3.5%) patients, loosening of one or more of osteosynthesis screws was observed (Figure 11, Figure 12, Figure 13 and Figure 14).

Malocclusion, which had to be corrected by orthodontic treatment, prosthetic treatment or a combination of those, occurred in 6 out of 113 (5.3%) patients. Almost 90% of the respondents did not have any complaints regarding the temporomandibular joint. In a further 11 of 113 (9.7%) patients, the transient ailments passed within 6 months of surgery.

The total rate of various complications in our study group was 25.7%. It means that 28 out of 113 operated patients developed one of the following complications: temporary hypofunction of the facial nerve, loosening of the screw, presence of occlusal disorders, presence of temporomandibular joint disorders.

## 5. Discussion

As already mentioned in the introduction to this paper, the qualification of patients for closed and open treatment is difficult and controversial [9,10,29]. In the study group, we performed surgical treatment in cases of occlusal disorders, comminuted fractures, dislocation within temporomandibular joint or lack of contact between bone fragments. Such rules are generally known and widely accepted [2,6]. The biggest qualifying problem is caused by cases where the fracture gap is single and the circumstances described above do not occur. In such situations, the criterion for qualifying for surgical treatment was shortening the mandible branch by 4 or more millimeters. However some authors suggest open treatment in cases of even smaller displacements, i.e., from 2 mm [30].

English literature lacks assessments based on large groups of patients regarding surgical treatment of fractures of the base and neck of the mandible condyle. To our knowledge, this study is the second largest, in terms of number of patients, indexed in the PubMed database of articles assessing the results of treatment using 3D plates. An exceptionally extensive study was conducted by Louvrier et al. on a group of 434 patients in the years 2006–2018 and published in 2020 [31]. The remaining similar studies from the last 10 years were conducted on 10 to 42 patients in the study group [7,15,32,33].

Among the patients from the aforementioned study, Louvrier et al. in 97.6% of cases use TCPs. In our material in 79.6% of cases it was decided to use DCCPs, and in 14.2% to TCPs. In other cases, more than one plate was used. We did not use the single straight or L-shaped plate technique knowing convincing arguments about the poor quality of such fixation [14,15]. According to clinical studies, the use of two miniplates gives good results, but it has a serious disadvantage of having to insert a minimum of 3–4 screws in the proximal bone fragment [14,15]. The use of a TCP allows insertion of 2 or more screws in a smaller fraction. Insertion of a larger number of screws is possible when a larger TCP size is used and depends on the size of the proximal fragment and bone quality. The difference between a small TCP and DCCP is due to the hole pattern for fixing the two screws. In the case of DCCP their arrangement is linear, in our opinion more suitable for stable fixation of the narrow neck of the mandible [7]. To prevent adverse mechanical factors resulting from the linear arrangement of the holes in the DCCP, a compression profiling of these holes was designed [7,15]. A common feature for various 3D plates shapes is their ease of adaptation to bone shape and application in place of bone fracture. According to our and other researchers, these features reduce the time of surgery [7,15].

Mandible abduction in our study group reached an average value of 49.2 mm and a median of 49 mm 6 months after surgery. No correlation was observed between this value and other examined factors. Considering the constant discussion on how to treat condyle fractures in cases of slight displacement, it is worth referring to the study of Asim et al. These authors observed that after 6 months, 40 patients treated surgically showed a statistically significant larger mouth opening by about 3 mm than 40 others treated with closed methods [34]. In the group of studies including 6-month observation or longer, average values of mouth opening after open treatment are given in the range from 36.4 to 44.0 mm [34,35,36]. These studies concerned various surgical approaches (including endoscopic), and various fixation materials. With this in mind, the greater range of mouth opening obtained in our material is probably related with early mobilizing patients jaw opening just after the surgery.

The authors of this work have not yet encountered the case of a 3D plate fracture in their practice. Other authors who tested 3D plates on animals and clinically also did not notice any fractures [7,15,37]. In turn, loosening of osteosynthesis screws occurs in patients treated with the use of one miniplate in approximately 13% to 19% of cases, and a higher percentage of this complication was observed for 3D plates [38,39]. These percentages were calculated for the groups where loosening of the screws even occurred. In the case of the large study group analyzed by us, this percentage is clearly lower, which may be a fair average between the groups without this complication and the above-mentioned ones. The percentage of patients with loose osteosynthesis screws was 3.5% in our material. Three out of four patients with loose screws were diagnosed with comminuted fractures, which may be related. In the case shown in Figure 13 and Figure 14, the proximity of the fracture gap could have caused the screw loosening. Zrounba et al. note that the likelihood of loosening of osteosynthesis screws increases in cases of higher fracture lines and bilateral fractures [40]. In our opinion, the right choice of 3D miniplate allows for proper placement of its holes on the bone surface, which reduces the percentage of loosening screws [7].

Ahuja et al. found directly after surgery 30% of occlusal disorders in a group of 10 patients treated with Delta plates and 40% in 10 patients in whom conventional mini plates were used. After a month, these authors obtained the correct occlusion in the entire 3D plates group. Malocclusion persisted in one patient in the group of conventional miniplates due to non-reduced concomitant fracture [15]. Pappachan et al. treated two groups of five patients using one and two L-shaped mini plates, respectively. Malocclusion after 3 months occurred in 60% only in the group treated with one mini-plate [14]. In the study of Yang et al. two of 36 (5.6%) patients surgically treated for fractures of the neck or condyle base had persistent occlusion disorders one year after surgery [41]. In our study, occlusal conditions were carefully monitored in the postoperative period. If required, a flexible maxillo-mandibular fixation was used. Despite this, in 5.3% of patients we were unable to achieve primary occlusion using surgical, orthopedic and functional therapy methods. In these cases, we ordered orthodontic or prosthetic treatment or a combination of them. When assessing the percentage of occlusal disorders in the material of other researchers, it should be taken into account that patients presenting e.g., comminuted fractures, other mandible fractures or non-mandible injuries were excluded from the studies referred to earlier, depending on their qualification criteria [14,15]. Our material involved 67.3% of patients with concomitant fractures of other parts of the mandible.

Among 113 patients included in our study, 13 (11.5%) reported complaints related to temporomandibular joints. In two patients (1.8%), temporomandibular disorders did not resolve within 6 months and had to be referred for further diagnosis and treatment. The first of these two patients had multiple mandible fractures and extensive trauma to other facial bones and soft tissues. Furthermore he was operated over 10 days after the injury. The second patient suffered from a multiple-site mandible fracture. Fracture of the condyle itself was comminuted, displaced and dislocated. In addition, the patient smoked over 20 cigarettes a day and often consumed alcohol. For comparison, Yang et al. report 5.6% and 19.4% respectively of pain and acoustic symptoms from the temporomandibular joints in a group of 36 patients examined one year after surgery [41]. After the same observation time, Yang and Patil noticed clicking in 8% of the 40 joints examined after open treatment of fractures of mandible condyles [36].

## 6. Conclusions

1. 3D titanium mini-plates are easy to adjust and take up little space, that’s why it can be widely used in cases of mandibular condyle fractures.

2. Stability of 3D osteosynthesis materials (no cases of plates breaking and low percentage of screws loosening) gives very good reliability for rigid fixation of bone fragments.

## Figures and Tables

**Table 1:** Criteria for including patients in the study.

Qualification Criteria
(1) Fracture in the anatomical region of the base and/or neck of the condyle of the mandible according to the AOCMF Classification System [2] (2) At least one of the following criteria: (a) presence of occlusal disorders (b) reduction of the vertical dimension of the mandible ramus by at least 4 mm due to displacement and overlapping of fragments (c) comminuted fracture (d) dislocation of the condyle in the temporomandibular joint (e) no contact between bone fragments (3) Age greater than or equal to 18 years

**Table 2:** Criteria for excluding patients from the study.

General Exclusion Criteria
(1) Lack of patient’s consent to open treatment (2) Contraindications for treatment under general anesthesia expressed in categories IV–VI on the ASA Physical Status Classification System scale
**Local Exclusion Criteria**
Mandibular head fracture according to the AOCMF Classification System [2]—the course of any fragment of the fracture line above the reference line of the head of the mandible

**Table 3:** Medical interview and examination form.

Patient Number	
Medical Interview	
Sex	Male/Female
Age	...
Age range	18–30/31–40/41–50/51–60/61–70/71–80
Residence	Village and town below 20 k/Medium town from 20 k to 100 k/Big city over 100 k
Tobacco use	Not applicable/Up to 10 cigarettes a day/11–20 cigarettes a day/More than 20 cigarettes a day
Alcohol consumption	Not applicable/Occasionally (less than once a week)/Frequently (1–3 times a week)/Alcohol dependence (more than 3 times a week)
Cause of injury	Violence effect/Fall and impact/Traffic accident/Sports accident/Others
Time from trauma to surgery, days	...
Time range from trauma to surgery	Up to 3 days/4–10 days/More than 10 days
**Clinical Examination**	
Classification of missing teeth according to Eichner [27]	Full dental arches (A1)/Supporting zones preserved (A2, A3)/Missing supporting zones (B1, B2, B3, B4, C1, C2)/Toothlessness (C3)
Coexisting mandibular fracture	Not applicable/Opposite condylar process/Mandibular angle/Mandibular body/Other mandibular fracture
Coexisting fracture of other facial bones	Not applicable/Zygomatic bone/Maxilla/Nasal skeleton/Other location/Fractures of multiple bones
Coexisting soft tissue injuries	Not applicable/Ecchymosis/Wound/Oedema/Combination of the above
Type of condylar fracture	Simple/Comminuted
Localization of the condylar fracture	Condylar base/Lower portion of the condylar neck
Displacement of bone fragments	No/Yes
Dislocation in the temporomandibular joint	No/Yes

**Table 4:** Characteristics of 3D plates used in the study.

4-DCCP	4-TCP	9-TCP
4-hole Delta Condyle Compression Plate	4-hole Trapezoid Condyle Plate	9-hole Trapezoid Condyle Plate
Medartis Modus 2.0 M-4894	Medartis Modus 2.0 M-4852 or M-4854	Medartis Modus 2.0 M-4858 or M-4860
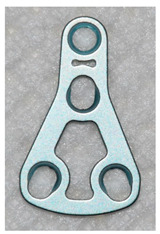	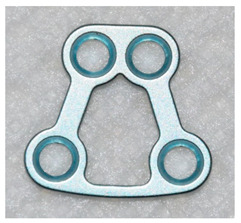	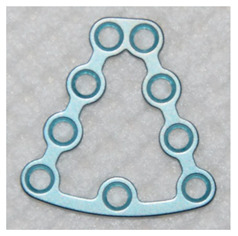
Material: Titanium ASTM F67 Elasticity: Semi-rigid Plate thickness: 1.0 mm		

**Table 5:** Observation form.

Hospitalization	
Surgical approach	Intraoral/Retromandibular transparotid/Submandibular
3D plate used	4-DCCP/4-TCP/9-TCP/more than one plate
Duration range of the operation	Less than 30 min/30–60 min/60–120 min/More than 120 min
Hospitalization time range	1–3 days/4–10 days/More than 10 days
**Outpatient Control After 6 Months**	
Assessment of facial nerve dysfunction according to House and Brackmann scale [28]	Normal function (I)/Mild dysfunction (II)/Moderate dysfunction (III)/Moderately severe dysfunction (IV)/Severe dysfunction (V)/Total paralysis (VI)
Interincisal mouth opening, mm	...
Plate breakage	Yes/No
Screw loosening	Yes/No
Malocclusion	Yes/No
Temporomandibular joints disorders (i.e., clicking)	Not applicable/Transient (up to 6 months)/Persistent (over 6 months)
